# Supplementary material for: Complex‐centric proteome profiling by SEC‐SWATH‐MS
Source: Mol Syst Biol. 2019 Jan 14;15(1):e8438. doi: 10.15252/msb.20188438 (PMC6346213; doi:10.15252/msb.20188438)
Supplement: Supplementary file 8 — Dataset EV7 [file MSB-15-e8438-s008.zip › feature_plots_string/P06733.pdf]

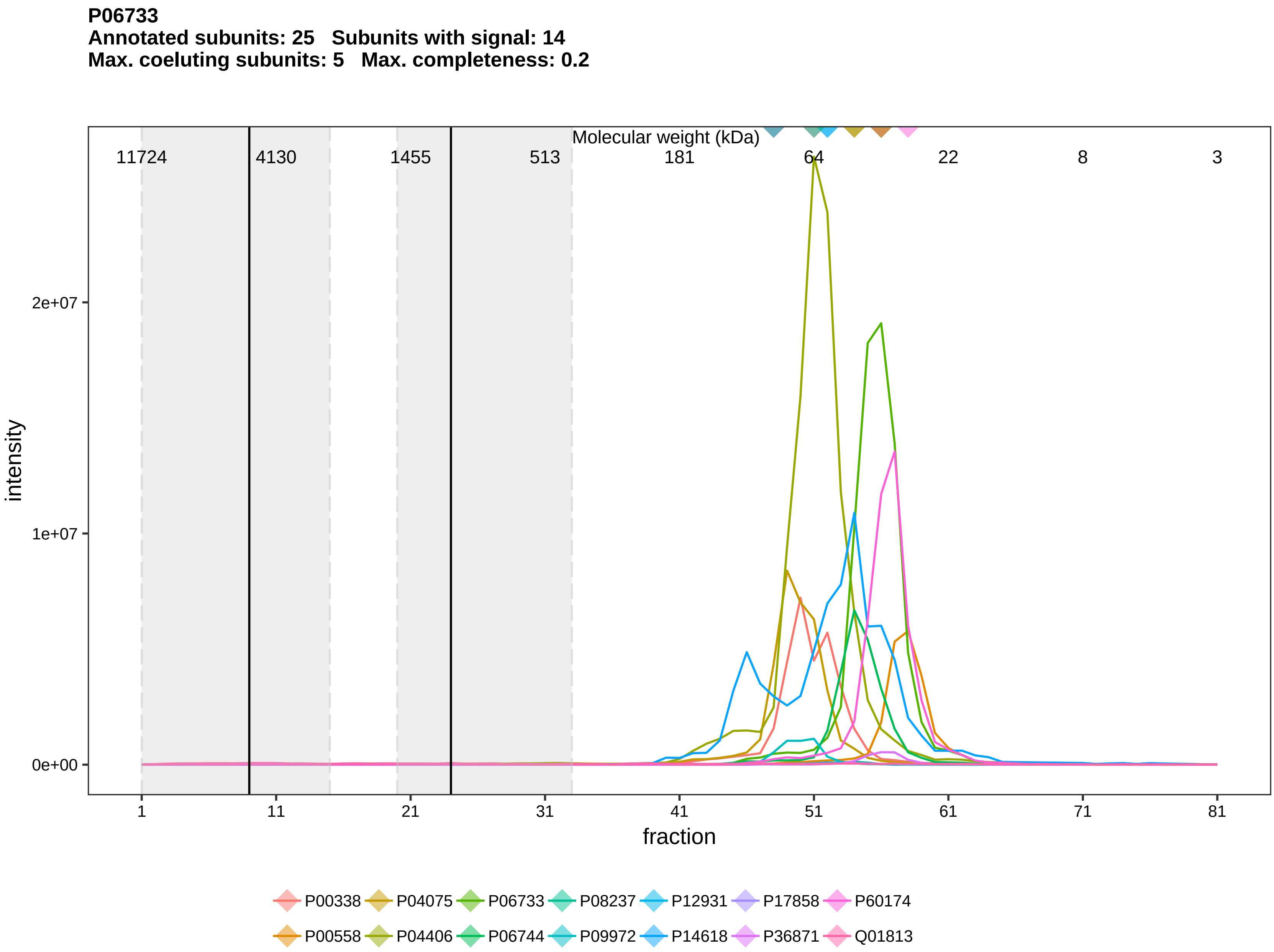

P06733
Annotated subunits: 25 Subunits with signal: 14
Max. coeluting subunits: 5 Max. completeness: 0.2
Molecular weight (kDa)
11724
4130
1455
513
181
64
22
8
3
intensity
2e+07
1e+07
0e+00
1
11
21
31
41
51
61
71
81
fraction
P00338
P04075
P06733
P08237
P12931
P17858
P60174
P00558
P04406
P06744
P09972
P14618
P36871
Q01813
